# First characterization of a microsporidial triosephosphate isomerase and the biochemical mechanisms of its inactivation to propose a new druggable target

**DOI:** 10.1038/s41598-018-26845-z

**Published:** 2018-06-05

**Authors:** Itzhel García-Torres, Ignacio De la Mora-De la Mora, Gloria Hernández-Alcántara, Dora Molina-Ortiz, Silvia Caballero-Salazar, Alfonso Olivos-García, Gabriela Nava, Gabriel López-Velázquez, Sergio Enríquez-Flores

**Affiliations:** 1 Grupo de Investigación en Biomoléculas, Laboratorio de Errores Innatos del Metabolismo y Tamiz, Instituto Nacional de Pediatría, Ciudad de México, 04530 Mexico; 2 Departamento de Bioquímica, Facultad de Medicina, Universidad Nacional Autónoma de México, Ciudad de México, 04510 Mexico; 3 Laboratorio de Toxicología Genética, Instituto Nacional de Pediatría, Ciudad de México, 04530 Mexico; 4 Laboratorio de Parasitología Experimental, Instituto Nacional de Pediatría, Ciudad de México, 04530 Mexico; 5 Unidad de Investigación en Medicina Experimental, Facultad de Medicina, Universidad Nacional Autónoma de México y Hospital General, Ciudad de México, 04510 Mexico; 6 Departamento de Microbiología y Parasitología, Facultad de Medicina, Universidad Nacional Autónoma de México, Ciudad de México, 04510 Mexico

## Abstract

The microsporidia are a large group of intracellular parasites with a broad range of hosts, including humans. *Encephalitozoon intestinalis* is the second microsporidia species most frequently associated with gastrointestinal disease in humans, especially immunocompromised or immunosuppressed individuals, including children and the elderly. The prevalence reported worldwide in these groups ranges from 0 to 60%. Currently, albendazole is most commonly used to treat microsporidiosis caused by *Encephalitozoon* species. However, the results of treatment are variable, and relapse can occur. Consequently, efforts are being directed toward identifying more effective drugs for treating microsporidiosis, and the study of new molecular targets appears promising. These parasites lack mitochondria, and oxidative phosphorylation therefore does not occur, which suggests the enzymes involved in glycolysis as potential drug targets. Here, we have for the first time characterized the glycolytic enzyme triosephosphate isomerase of *E. intestinalis* at the functional and structural levels. Our results demonstrate the mechanisms of inactivation of this enzyme by thiol-reactive compounds. The most striking result of this study is the demonstration that established safe drugs such as omeprazole, rabeprazole and sulbutiamine can effectively inactivate this microsporidial enzyme and might be considered as potential drugs for treating this important disease.

## Introduction

Microsporidia is a group of spore-forming intracellular parasites that infect a large number of vertebrate and invertebrate hosts^1^. For the last three decades, reports of microsporidia infections have increased significantly, mainly in immunocompromised people diagnosed with HIV/AIDS^2^, organ transplant recipients, bone marrow graft recipients, chemotherapy patients, traveling people, and the pediatric population and elderly people^3,4^. Thus, the microsporidia are considered to be of worldwide clinical importance.

This group consists of approximately 1400 species, 12 of which have been frequently reported in humans, including *Enterocytozoon bieneusi*, *Encephalitozoon intestinalis*, *E. cuniculi* and *E. hellem*^5^. *E*. *bieneusi* and *E*. *intestinalis* are the most common microsporidia^6^.

Worldwide, the reports of infections in the previously mentioned populations vary from 0 to 60%. For example, reports indicate a range of prevalence in Europe from 2.5 to 42%, whereas in Asia varies from 1.38 to 13%, and in Africa varies from 5.2 to 58.1%^6^. The above data depend on several factors such as geographic region, the diagnostic method employed, and the studied population^7^. In America the prevalence ranges from 1.5% (USA) to 60%, being Mexico the country with the highest prevalence reported^8–11^.

The transmission of microsporidia occurs primarily by the fecal-oral route, the sources of infection can include water or food contamination; on the other hand, transmission by contact with animals has been strongly suggested^12^ and several studies have confirmed this hypothesis^13^. When humans are infected, the spores (infective form) usually travel through the small intestine and they extrude the polar tube, which contacts the host cell to transmit its sporoplasm^14^, initiating the life cycle. Within the cell, the sporoplasm gives rise to the meronts (proliferative phase or merogony), which multiply; the second phase (maturation phase or sporogony) grows and develops to sporoblasts, sporonts and finally the spores. Later, the host cell is lysed, releasing the spores to the surroundings, and can infect adjacent host cells or can be transported in feces, urine, secretions of the respiratory system and bodily fluids. Normally, microsporidiosis causes constant diarrhea, malabsorption accompanied by progressive weight loss, nausea, vomiting and severe dehydration^15^. However, if other organs are invaded, the symptoms will correspond to the specific location^16^. For example, conditions have been reported to include hepatitis, sinusitis, ocular infection and brain infection (encephalitis), which in severe cases can cause the death of the patient^17^.

When microsporidiosis is diagnosed, albendazole and less frequently fumagillin are prescribed^6^. Both drugs are administered for several weeks, and the doses vary according to the specific characteristics of the disease^18^. Importantly, fumagillin has been demonstrated to cause adverse effects such as thrombocytopenia, neutropenia and hyperlipidemia when administered systemically in humans^6^. On the other hand, because the family of benzimidazoles have been used for more than 30 years against many parasitic organisms, resistance has been demonstrated in diverse species including the microsporidia group^19^. Accordingly, *E*. *intestinalis* resistance is suggested to be due to the presence of two multidrug resistance genes^20,21^.

Therefore, the search for new treatments for microsporidiosis caused by one of the most frequent microsporidia, *E*. *intestinalis*, is crucial. Proteins belonging to energetic pathways are attractive therapeutic targets, and *E*. *intestinalis* notably lacks organelles that produce energy in the form of ATP, such as mitochondria^22^. In contrast, glycolysis is a well conserved route in microsporidia, which is of special interest since all the genes of this energetic pathway have been observed in the genomes of microsporidia (including *E. intestinalis*). Reinforcing the above, glycolytic activity has been reported^23^ and has been demonstrated to generate ATP and suggested to generate metabolites for other pathways^24^.

An important and well-known glycolytic enzyme is the triosephosphate isomerase (TIM) [EC 5.3.1.1] that catalyzes the reversible interconversion of the isomers dihydroxyacetone phosphate (DHAP) and D-glyceraldehyde 3-phosphate (GAP). The kinetic parameters of most TIMs have been studied, and the crystallographic structures of approximately 199 TIMs are available in the Protein Data Bank^25^. Additionally, TIMs of parasitic organisms such as *Trypanosoma cruzi*^26^, *T. brucei*^27^, *Entamoeba histolytica*^28^, *Giardia duodenalis*^29^, and others have been studied and proposed as targets to develop new pharmacological compounds. These reports have shown that the chemical modification (derivatization) of the cysteine (Cys) residues of such enzymes by sulfhydryl reagents leads to severe alterations at the functional and the structural levels. Moreover, these studies indicate that the general mechanism of inactivation could be considered for drug design; on this basis, some candidate molecules have been proposed as either prodrugs^26^ or drugs^29^. Moreover, new uses for approved and safe drugs have been proposed to make the process of drug design more efficient. Nonetheless, until now there are no approved antiparasitic drugs which mechanism of action is through derivatization of Cys residues. Considering all the factors mentioned above, the TIM of *E*. *intestinalis* (EiTIM) could be a good target for drug design.

In this work, we characterized the TIM from the human parasite *E. intestinalis* (EiTIM) as a potential therapeutic target.

## Results

### The gene for triosephosphate isomerase of *E. intestinalis* exhibits the general characteristics of the TIMs

**Figure 1:**
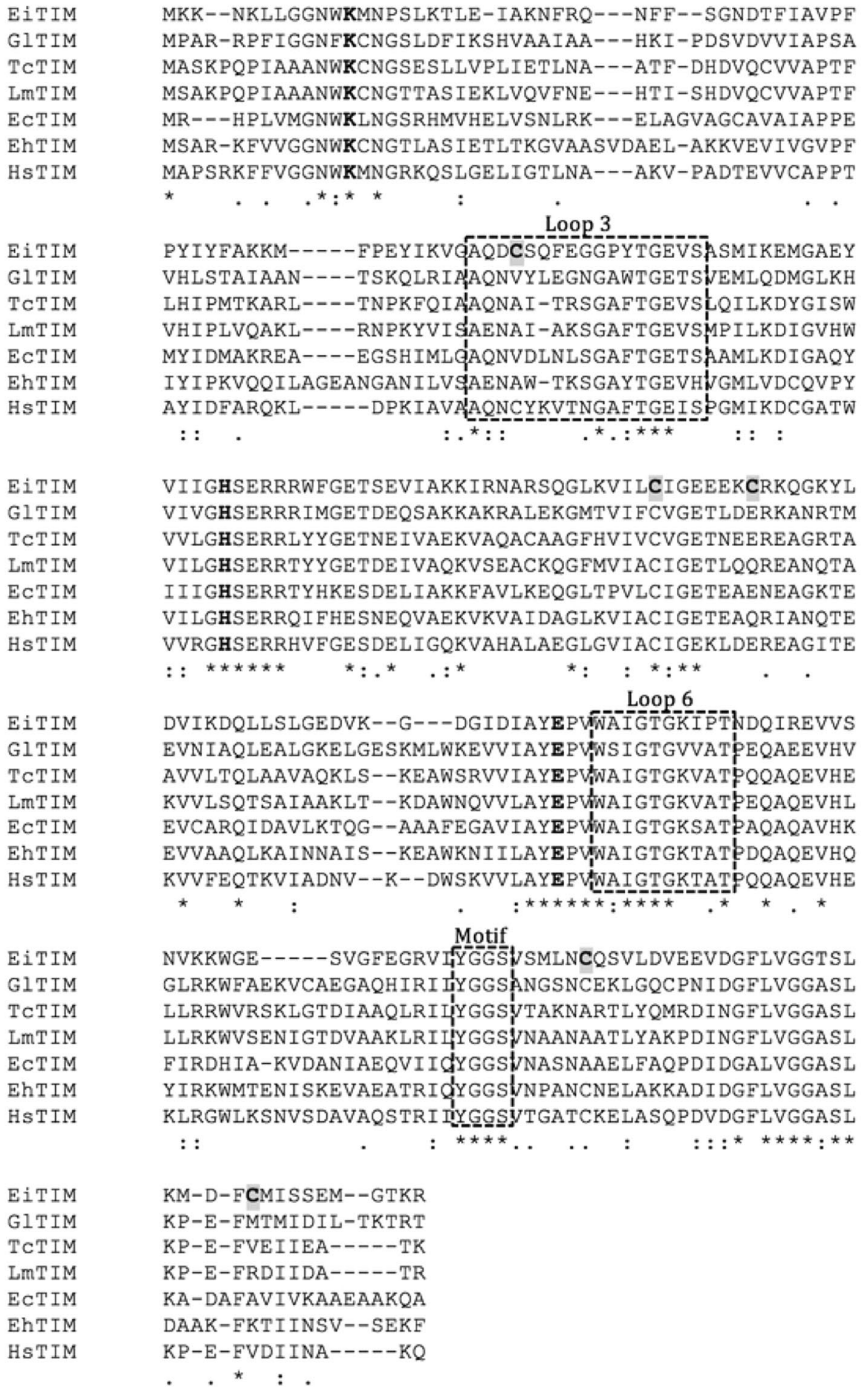
Sequence alignment of various TIMs. The sequence alignment of TIMs from *E. intestinalis* (EiTIM), *Giardia duodenalis* (GlTIM), *Trypanosoma c*ruzi (TcTIM), *Leishmania mexicana* (LmTIM), *Escherichia coli* (EcTIM), *Entamoeba histolytica* (EhTIM) and *Homo sapiens* (HsTIM). The active site residues are in bold; loops 3, 6 and the conserved motif are in a dashed-line box. The Cys residues of EiTIM are shaded in gray. The alignment was performed with Clustal W (1.81) (http://www.clustal.org/).

The nucleotide sequence of the gene *eitim* was obtained from the NCBI database (accession number XM_003073826.1), and the corresponding amino acid sequence was analyzed for the conserved (canonical) amino acid residues, motifs and domains reported for other TIMs. Previous studies on several TIMs have reported strictly or moderately conserved amino acids that belong to the active site and to motifs or loops that are involved in the catalytic activity, substrate incorporation, and dimerization, among other functions^25^. By sequence alignment analysis, such amino acids can easily be identified in EiTIM (Fig. 1). For example, the catalytic residues lysine, histidine and glutamic acid are located at positions 12, 92 and 161, respectively (according to the EiTIM sequence numbering) (Fig. 1, bold letter). Other conserved and well-described residues, such as those reported to be involved in the correct incorporation of the substrate (TyrGlyGlySer motif)^30^, can be similarly identified (Fig. 1). Importantly, this motif has been observed to interact with the catalytic loop (loop 6), which is formed by the amino acid residues 166 to 173 and has been described to function as a lid that opens and closes to exclude water molecules located at the active site, facilitating enzymatic catalysis^31^ (Fig. 1). Another structurally important region in TIMs is the moderately conserved interdigitating loop (loop 3), which consists of amino acids that interact with the adjacent monomer to form the biological unit, the homodimer. This loop corresponds to amino acid residues 60 to 76 in the EiTIM sequence (Fig. 1). Finally, five Cys residues are distributed throughout the EiTIM sequence, of which only one is conserved (Cys 123), while the other four occur less frequently (Fig. 1).

The presence of these biologically important regions of TIMs in the EiTIM amino acid sequence indicates that this protein exhibits the general characteristics of such glycolytic enzymes, which would allow it to carry out the main enzymatic function of TIMs. Additionally, we took advantage of the global distribution and low conservation of the Cys residues to explore the possible inactivation of this enzyme by the chemical modification of these amino acid residues.

### Triosephosphate isomerase of *E. intestinalis* is a catalytically competent enzyme

The *eitim* gene was cloned into a pET3a-HisTEV modified plasmid, and the corresponding soluble protein was successfully over-expressed in *E. coli* BL21 DE3p*Lys*S cells. The recombinant EiTIM was purified in a single step by using IMAC-nickel resin, followed by removal of the His-tag sequence. SDS-PAGE analysis showed the high purity of the recombinant enzyme (Supplementary Fig. S1): lane 2 shows a single distinctive band with a molecular mass of ~27 kDa. This result is consistent with the size of the EiTIM monomer (27.03 kDa). Additionally, the purification yield was 5 mg L^−1^ of LB culture, and the purity was greater than 95%, as determined by densitometry (data not shown).

One notable characteristic of the TIMs is their high catalytic activity in interconverting the substrates DHAP and GAP. Thus, experiments were carefully conducted to determine the specific activity of EiTIM with GAP as the substrate. The results showed that the recombinant enzyme has a specific activity of 1100 μmol min^−1^ mg^−1^ ± 125. This value is within the range of TIM activities reported in the Enzyme Database - BRENDA (https://www.brenda-enzymes.org/), which can vary from a few to several thousands of units.

To obtain the kinetic parameters, the initial velocities of the enzyme were measured at different substrate concentrations, and the values were plotted as a function of the GAP concentration. The results indicate that the Michaelis-Menten model fit the graph well (Supplementary Fig. S2, solid red line). Thus, we estimated the kinetic parameters: *V*_max_ was 1874 μmol min^−1^ mg^−1^ ± 180, and *K*_m_ was 0.83 mM ± 0.2 (Table 1).

**Table 1:** Kinetic properties of TIMs from diverse parasites and human.

Enzyme	*K*_m_ (mM)	*k*_cat_ (min^−1^)	*k*_cat_/*K*_m_ (M^−1^ s^−1^)	Ref.
EiTIM	0.83 ± 0.20^*^	1.01 × 10^5^ ± 3.17^*^	2 × 10^6^ ± 5.74^*^	This work
TcTIM	0.43	2.7 × 10^5^	1.05 × 10^7^	García-Torres *et al*.^54^
EhTIM	0.61	2.41 × 10^5^	6.5 × 10^6^	Landa *et al*.^55^
GlTIM	0.78	4.6 × 10^5^	9.8 × 10^6^	Enríquez-Flores *et al*.^48^
HsTIM	0.74	1.85 × 10^5^	4.2 × 10^6^	De la Mora-De la Mora *et al*.^56^

^*^These data represent the mean of four independent experiments.

These results demonstrate that the enzymatic activity of EiTIM has a canonical Michaelis-Menten saturation curve and shows affinity for GAP, the substrate widely used to determine TIM activity. In addition, the *K*_m_ and *k*_cat_ values obtained for EiTIM are consistent with data reported in studies involving TIMs from other species (Table 1).

### The secondary structure of EiTIM is a canonical and well-folded TIM barrel

**Figure 2:**
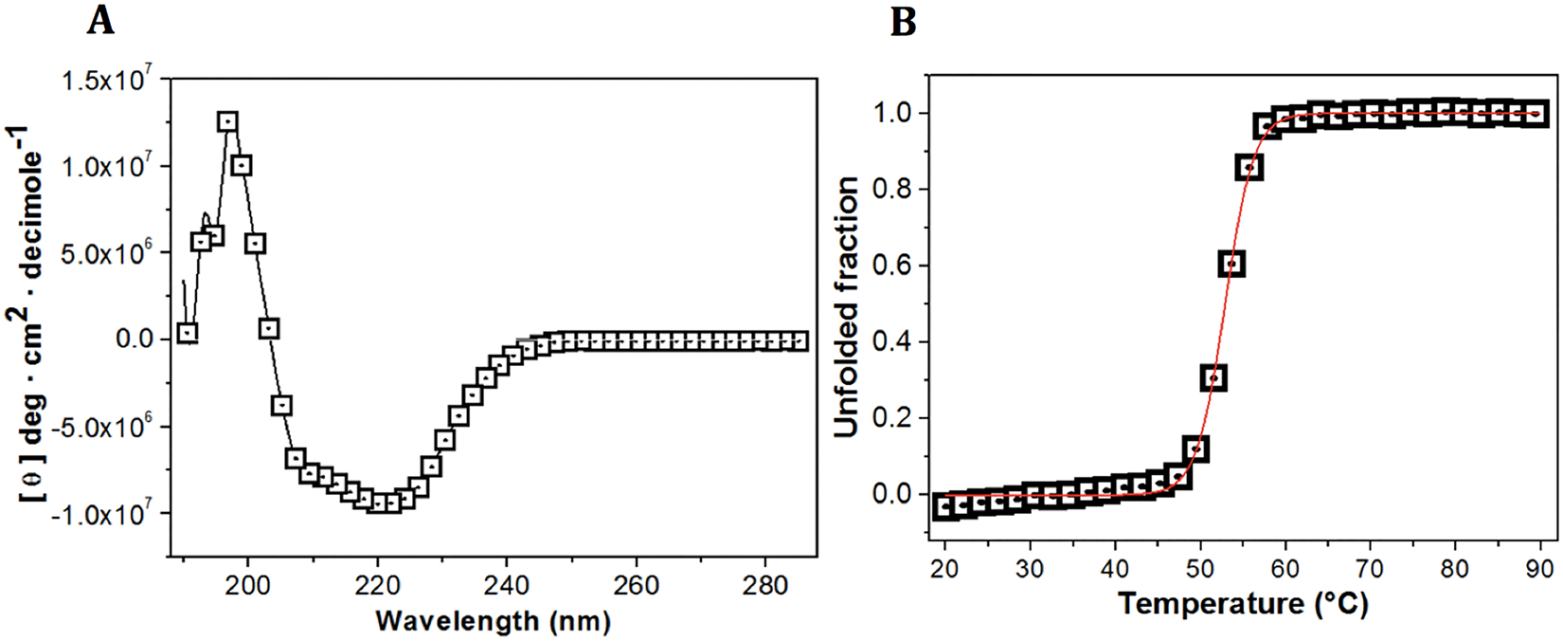
Spectroscopic properties of EiTIM. Far-UV CD spectra (**A**) and thermal denaturation of EiTIM (**B**). The CD spectrum (280 to 190 nm) was measured with 0.5 mg mL^−1^ of the protein dissolved in TE buffer at 25 °C (**A**), and the denaturation curve was measured at 222 nm with increases in temperature (**B**). The data are the mean of at least four independent experiments.

The structural characteristics and folding properties of EiTIM were determined by CD in the far-UV region. Figure 2A shows the CD signal of EiTIM. The spectrum is well defined with minimum peaks at 208, 210 and 222 nm. This CD pattern has been commonly reported for several TIMs and corresponds to the signal of a protein with the same content of alpha helices and beta sheets. The spectroscopic characteristics of EiTIM are consistent with those of a beta-barrel conformation. Therefore, the results demonstrate that the EiTIM is a well-folded protein with secondary structure like that of a canonical TIM barrel.

To establish the melting temperature (Tm) of EiTIM, ellipticity changes were followed at 222 nm with temperature increases. In the denaturation profile, the protein remained at its baseline (native structure) until approximately 45 °C (Fig. 2B), and above this temperature, the enzyme was denatured cooperatively, completing the process at approximately 60 °C. The calculated Tm was 52.8 °C (Fig. 2B, red line), which is slightly lower than the values reported for other TIM enzymes (Table 2).

**Table 2:** Melting temperature (Tm) of EiTIM and other TIMs.

TIM	Tm (°C)	Ref.
EiTIM	52.8 ± 1.5^*^	This work
TcTIM	57.3	Unpublished data
EhTIM	58.6	Landa *et al*.^55^
GlTIM	57.5	Enríquez-Flores *et al*.^32^
HsTIM	61.2	De la Mora-De la Mora *et al*.^56^

^*^These data represent the mean of four independent experiments.

### EiTIM is efficiently inactivated by sulfhydryl reagents

**Figure 3:**
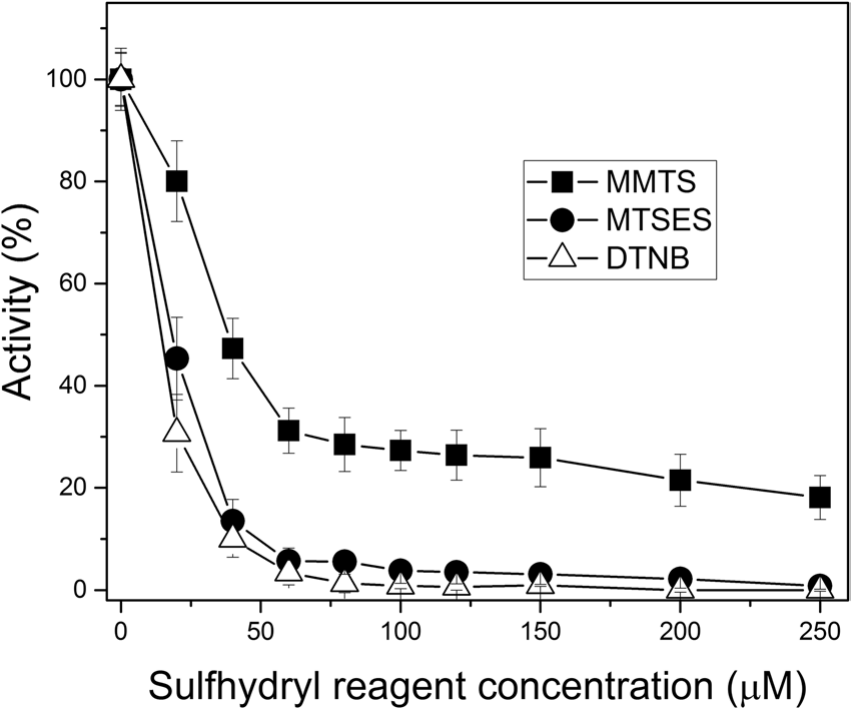
Inactivation of EiTIM by three different sulfhydryl reagents. The protein was incubated with MMTS (closed squares), MTSES (closed circles) or DTNB (open triangles). After 2 h of incubation at 37 °C, an aliquot was withdrawn, and the residual activity was monitored as reported in Materials and Methods. The data are the mean of at least four independent experiments.

In this work, we examined the inactivation of EiTIM via the derivatization of its Cys residues in order to investigate the protein’s potential as a target for drug design. For this purpose, the protein was incubated in the presence of three sulfhydryl reagents (MMTS, MTSES and DTNB) at increasing concentrations, and the enzymatic activity was determined after an incubation period. In the case of inactivation of EiTIM by MMTS, Fig. 3 (closed squares) shows a 60% decrease in EiTIM activity at 50 μM of this sulfhydryl reagent. Above this concentration, no significant changes were observed, and the protein retained its remaining enzymatic activity. Importantly, although 80% inactivation was observed, total inactivation was not obtained at any MMTS concentration. To improve the inactivation, the sulfhydryl reagents MTSES and DTNB were used (Fig. 3, closed circles and open triangles, respectively). As shown, the inactivation process was very similar for both reagents: the enzymatic activity decreased by more than 95% at concentrations lower than 60 μM, and at higher concentrations, EiTIM was totally inactivated. Thus, these sulfhydryl reagents successfully inactivated EiTIM in a concentration-dependent manner. The physicochemical characteristics of the probes are of interest due to the differences in inactivation.

Importantly, the sulfhydryl reagents employed here (MMTS, MTSES and DTNB) exhibit no significant effect on the activity of human triosephosphate isomerase (HsTIM), as previously reported for MMTS and DTNB^32^, and as described in this work for MTSES (Supplementary Fig. S3).

Taking these results together, we have demonstrated that EiTIM can be wholly and efficiently inactivated via the derivatization of Cys residues by sulfhydryl reagents.

To understand the inactivation mechanism, we determined the number of derivatized Cys residues in EiTIM. Table 3 shows the results for the enzyme in the absence or in the presence of the sulfhydryl reagents (control and derivatized enzyme, respectively). In denaturing conditions, the number of free Cys residues (per subunit) of the control enzyme was 5, which is consistent with the number of Cys residues in its amino acid sequence (Fig. 1, gray shaded letter). In contrast, the assays showed that the number of free Cys residues per subunit of the derivatized enzymes was near 2. According to these results, given that the total number of Cys residues per EiTIM subunit is 5, our results indicate that 3 Cys residues per subunit were derivatized by the sulfhydryl reagents. Additionally, the activity of the enzyme under these conditions was measured, indicating that it reached its maximum inactivation (Fig. 3 and Table 3). Thus, these results demonstrate that the derivatization of at least 3 Cys per subunit in the EiTIM leads to a partial (MMTS) or total (MTSES and DTNB) loss of enzymatic activity.

**Table 3:** Quantification of the free Cys residues of EiTIM under non-derivatizing (control) and derivatizing conditions.

EiTIM	Free Cys/subunit^*^	Derivatized Cys/subunit	Activity (%)^*^
Control	4.9 ± 0.3	0	100
+ MMTS	2.1 ± 0.2	3	17.3 + 5.5
+ MTSES	2.0 ± 0.3	3	2.4 + 1.1
+ DTNB	1.8 ± 0.2	3	0.5 ± 0.3

^*^These data represent the mean of four independent experiments.

### The EiTIM inactivated by sulfhydryl reagents is drastically altered at the structural level

Our data reveal that the sulfhydryl reagents strongly affect EiTIM at the functional level; therefore, determining the potential changes at the structural level upon maximum inactivation of the enzyme was of considerable interest. Thus, we measured the CD spectrum and Tm under inactivation conditions. The results indicate that the secondary structure revealed by CD of the inactivated enzymes is similar to that of the control enzyme (Supplementary Fig. S4), so the enzyme was not significantly affected at this level. Interestingly, however, the Tm values of the derivatized enzymes were lower than that of the control enzyme (Table 4, and supplementary Fig. S5): according to these data, the Tm decreased by approximately 3 to 5 °C in comparison with that of the control enzyme. Therefore, the global stability of EiTIM was significantly affected by inactivation with the sulfhydryl reagents.

**Table 4:** Tm values of EiTIM under non-derivatizing (control) or derivatizing conditions. Additionally, the residual activity was measured under each condition.

EiTIM	Tm (°C)^*^	Activity (%)^*^
Control	52.8 ± 1.5	100
+ MMTS	49.8 ± 2.1	17.3 ± 5.5
+ MTSES	48.6 ± 1.9	2.4 ± 1.1
+ DTNB	47.9 ± 2.2	0.5 ± 0.7

^*^These data represent the mean of four independent experiments.

In other assays, the structural alterations were further evaluated by the binding of ANS. ANS is a molecule with high affinity to hydrophobic areas in proteins, leading to a strong fluorescence emission that can be monitored. Thus, the fluorescence emission spectra of ANS in the presence of non-derivatized and derivatized enzyme were determined.

**Figure 4:**
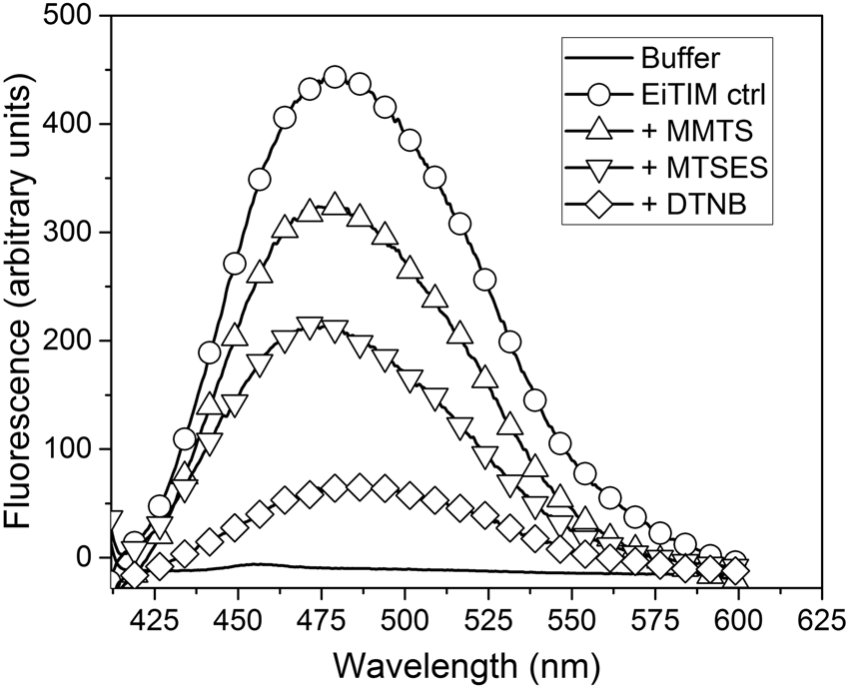
Fluorescence spectra of ANS in the presence of non-derivatized or derivatized EiTIM. After exposure to EiTIM in the absence or presence of the derivatizing sulfhydryl reagents, the fluorescence of ANS was measured with excitation at 395 nm. The ANS without enzyme was also measured (in TE buffer), the background signal was subtracted. The data are the mean of at least four independent experiments.

Figure 4 shows the fluorescence emission spectra of ANS corresponding to the control and derivatized enzymes. As observed, the highest fluorescence intensity was obtained for the non-derivatized enzyme, with a maximum signal of 446 (a.u.), corresponding to a wavelength of 478.5 nm (Fig. 4, open circles). For the EiTIM derivatized with sulfhydryl reagents, the maximum signals were recorded at 323, 214 and 66 (a.u.), corresponding to wavelengths of 479.5 nm, 476 nm and 484 nm (Fig. 4, + MMTS, + MTSES and + DTNB, respectively).

In general, the fluorescence of the inactivated enzymes decreased. With the fluorescence intensity for the control taken to be 100%, the fluorescence intensity for the inactivated enzymes was 72.5, 48.0 and 14.8% for + MMTS, + MTSES and + DTNB, respectively. Interestingly, this behavior corresponded to the respective degrees of inactivation. This notable decrease in fluorescence intensity suggests that the hydrophobic regions of the derivatized enzyme decreased drastically, indicating significant structural alteration in the presence of the sulfhydryl reagents.

### Effects of different commercially approved drugs on the inactivation of EiTIM via derivatization of its Cys residues

**Figure 5:**
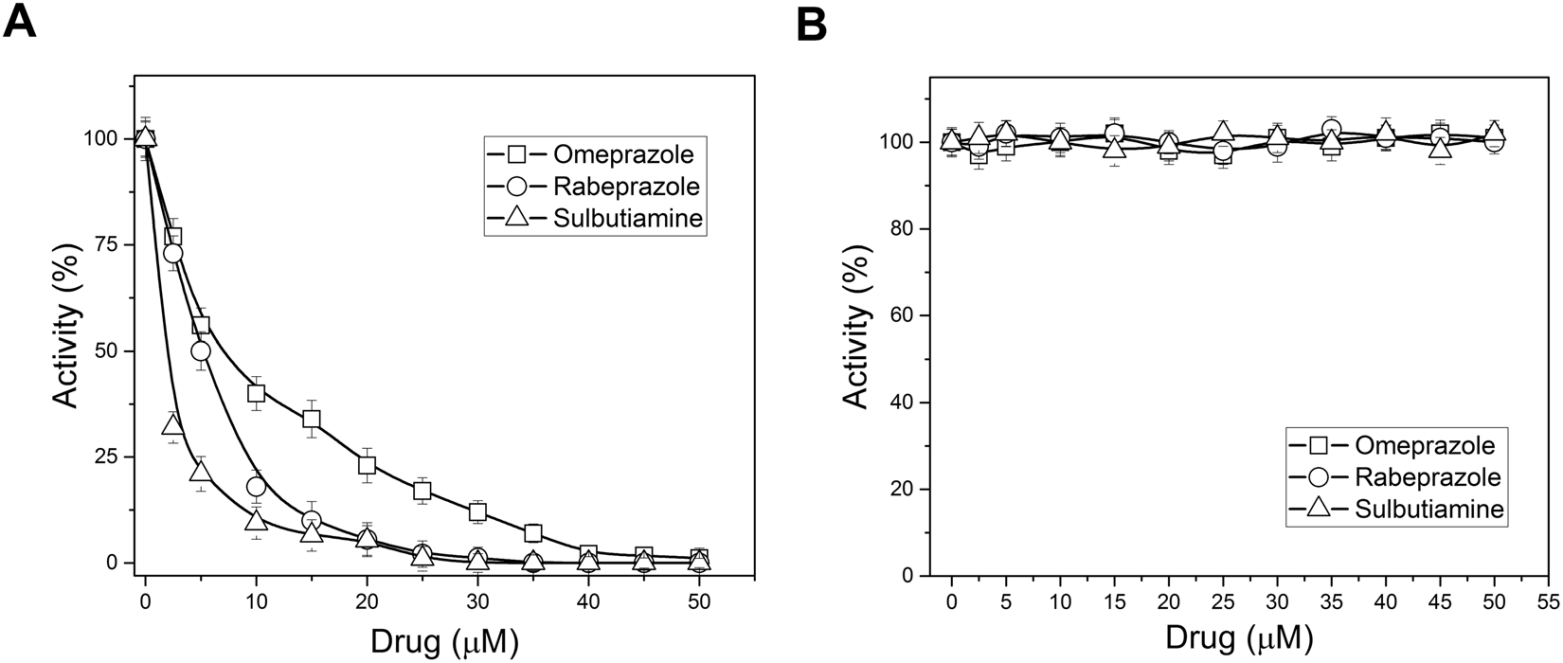
Derivatization of EiTIM and HsTIM with drugs. The enzymes [(**A**) EiTIM and (**B**) HsTIM] were incubated at increasing concentrations of omeprazole (open squares), rabeprazole (open circles) and sulbutiamine (open triangles). After 2 h of incubation at 37 °C, an aliquot was withdrawn, and the residual activity was monitored as reported in Materials and Methods. The data are the mean of at least four independent experiments.

In our efforts to find a potential drug that would inactivate this enzyme, we focused on some commercially approved drugs that could chemically modify Cys residues. We chose two proton pump inhibitors (PPIs), omeprazole and rabeprazole, and sulbutiamine, which is an analogue of vitamin B, to perform EiTIM inactivation assays. The PPIs are widely used for the treatment of gastric and duodenal ulcers, while sulbutiamine is useful in alleviating fatigue. This latter molecule has been reported as an enhanced version of vitamin B1 with several benefits for reducing fatigue^33^. In fact, many reports show that these commercial drugs are safe in humans, and their mechanism of action is the chemical modification of Cys residues^29,34,35^. The assays consisted of exposing the enzyme to increasing concentrations of the drugs, and after an incubation period, the residual enzymatic activity was measured. Interestingly, the activity of EiTIM was totally abolished by all the drugs (Fig. 5A). Of the drugs, sulbutiamine inactivated the enzyme most effectively, requiring low concentrations of approximately 10 μM to achieve the total inactivation of EiTIM. In terms of 50% inactivation, sulbutiamine was two- and three-fold more effective than rabeprazole and omeprazole, respectively. Notably, sulbutiamine was more effective than the sulfhydryl reagents assayed in this work. For example, 2.5 μM sulbutiamine was required to achieve 50% inactivation, whereas the concentration of DTNB required to obtain the same percentage inactivation was 25 μM (ten-fold). Finally, it is important to mention that these drugs do not significantly affect the counterpart HsTIM (Fig. 5B)^29^.

## Discussion

*E. intestinalis* is an organism that has evolved toward the reduction or loss of subcellular components, metabolic pathways and proteins^36^, due in part to its parasitic lifestyle. Reinforcing the above characteristics, its genome is known to be the smallest among eukaryotic organisms (2.3 megabase pairs)^37^. Notwithstanding, this organism retains some principal metabolic pathways for carbon metabolism, such as the pentose phosphate pathway, the trehalose metabolism and the glycolytic pathway^24^. The evidence is strengthened by the demonstration that this parasite contains mitochondrial relics that do not perform oxidative phosphorylation and therefore lacks the tricarboxylic acid cycle^22,38^, which strongly suggests that the core carbon pathways are highly relevant for the survival of this parasitic organism.

On the other hand, although the expression of the glycolytic enzymes in *E. intestinalis* has not been demonstrated to date, studies have been conducted in closely related microsporidia such as *E. cuniculi*, *Nosema gryllii, N. bombycis*, and *Trachipleistophora hominis*, among others, in which the presence and enzymatic activity of such proteins has been well defined^23,39,40^. Therefore, this same situation is strongly suggested to occur in *E*. *intestinalis* due to the above points and the fact that the complete amino acid sequences of all the glycolytic enzymes have been already identified (microsporidiadb.org/micro/).

Due to the dependence on the host, microsporidia have been reported to lack the ability to use glycolysis as a continuous source of energy, since ATP can also be obtained from the host. This ability is particularly important in *E. intestinalis* since the supply of such energetic molecules by the host does not mean that glycolysis is inactive. The glycolytic route has been suggested to play an important role in the supply of metabolites necessary for the pentose phosphate and trehalose pathways^24^. Therefore, glycolysis might provide not only energetic molecules but also other metabolites that feed the diverse metabolic pathways necessary for the physiological processes of this organism.

Due to the above, we characterized the triosephosphate isomerase of *E. intestinalis* with the aim of proposing it as a new potential target of pharmacological compounds. This work is the first report of the characterization of a glycolytic enzyme belonging to this intracellular parasite. Our results demonstrate that EiTIM is an enzyme whose amino acid sequence, catalytic characteristics and structural properties are consistent with those of previously reported typical TIMs. This evidence allows us to conclude that the nucleotide sequence of the *eitim* gene reported in the NCBI corresponds to an enzyme with triosephosphate isomerase activity.

With the objective of proposing this enzyme as a new and promising pharmacological target, we evaluated the inactivation of EiTIM by sulfhydryl reagents. Our results indicate that it is indeed possible to inactivate this enzyme by the chemical modification of one to three Cys residues. On the basis of the results, we also suggest that the total inactivation could be dependent on the physicochemical characteristics of the compounds.

The data presented here are in agreement with those in other studies that have shown the inactivation of enzymes in other parasites by the derivatization of Cys residues^32,41,42^. Such studies have demonstrated that the derivatization leads to inactivation, and the mechanism of action depends on which Cys is derivatized (*i.e*., dimer dissociation or the structural alteration of loops)^41,43^. In this regard, our studies show that three Cys residues are involved in the inactivation process, and according their positions show equivalence to Cys residues in other TIMs. For example, Cys 123 of EiTIM (127 in most TIMs) is a strictly conserved amino acid, and its presence has been related to the correct folding of the TIMs^44^. However, the derivatization of this Cys under native conditions has not been reported to date, so this Cys is unlikely to be involved in the inactivation. Cys 208 of EiTIM is equivalent to Cys 222 of GlTIM, and the chemical modification of Cys 222 has been demonstrated to induce inactivation and drastic perturbation at the structural level^43^. In contrast, Cys 63, 130 and 232 have no equivalents in others TIMs. Most likely, some of these Cys are involved in the inactivation of EiTIM, so further studies are needed to elucidate the detailed mechanism of inactivation.

It was also observed that the inactivated EiTIM loses structural stability at the global level (Table 4) and drastic changes in the binding for ANS (Fig. 4). These alterations demonstrate that the damage extended beyond interference with enzyme catalysis. Similarly, the Tm of GlTIM inactivated by sulfhydryl reagents has been shown to decrease drastically^32^.

The Cys residues in EiTIM are located in non-conserved sites, and some previous reports have shown that disturbances at sites other than conserved sites, such as active sites, can lead to losses in structural stability^42^. This result is highly important, since poorly preserved sites at which the enzyme is inactivated or destabilized can be good targets for drug design. Such is the case of TcTIM and GlTIM, among others, where the perturbation of Cys residues near the interface or distant from the active site results in severe effects at the functional and structural level. Therefore, our results demonstrate that EiTIM can be proposed as a target for compounds with pharmacological potential via the derivatization of Cys residues. Supporting this assertion, as mentioned, HsTIM was not significantly affected by exposure to these sulfhydryl reagents, despite the presence of 5 Cys residues in its amino acid sequence.

Going a step further in the search for compounds that could be proposed as potential drug candidates, we evaluated the inactivation of EiTIM with three drugs that are already widely studied and used in the pharmaceutical industry. This work involves applying the knowledge of a general mechanism of inactivation, in this case, the chemical modification of Cys residues, to the proposal of new uses for commercially approved and safe drugs. We propose two PPIs, omeprazole and rabeprazole, and a nootropic, sulbutiamine. Our results clearly demonstrate that EiTIM is efficiently inactivated by these drugs. Because the mechanism of these drugs has been reported to be the derivatization of Cys residues, we suggest that the mechanism of inactivation is similar to that observed with the sulfhydryl reagents. Relatedly, the exposure of GlTIM to omeprazole and its derivatives has recently been shown to lead to efficient inactivation of this enzyme^29^. Sulbutiamine is a molecule consisting of two thiamin (B1) molecules bound together by a disulfide and is a lipophilic drug that is useful in alleviating fatigue^35^; its efficacy in the aforementioned and safety in humans have been previously demonstrated. From our discovery on the sulbutiamine action over *E. intestinalis* TIM, it should be good to assay this drug against other parasitic TIMs. The most attractive feature of the sulbutiamine to our work is that it is a lipophilic drug, which crosses the blood-brain barrier; therefore, is a promising drug in the treatment of brain microsporidiosis (encephalitis).

The fact possibility of inactivating EiTIM could result in severe consequences for these microsporidia. In other organisms, alterations to this enzyme that cause its malfunction (mutagenesis, inactivation with molecules, gene deletion, etc.) are detrimental to the cell^45,46^. For example, when DHAP accumulates, it is spontaneously converted to methylglyoxal, which in turn is a toxic molecule that damages the cell. In many organisms, the existence of detoxification systems (glyoxalase system) has been demonstrated^47^; however, the presence of this detoxifying system in *E*. *intestinalis* has not been demonstrated to date, and no such genes are reported in the genome of this organism. Therefore, EiTIM inactivation should lead to an accumulation of DHAP and to the generation of methylglyoxal, which could cause cellular toxicity. In conclusion, this work shows the first functional and structural characterization of a microsporidial glycolytic enzyme, and presents a breakthrough in the development of new anti-*Encephalitozoon* compounds.

Further studies assaying the compounds and drugs studied here against the TIMs from other species of *Encephalitozoon* will support our proposal. To corroborate our hypothesis it is necessary to demonstrate the effectiveness of the studied drugs as anti-*Encephalitozoon* drugs in axenic parasite cultures, and the demonstration in animal models would be an important step to propose an innovative method in the treatment of microsporidiosis.

## Materials and Methods

### Reagents and general materials

Unless otherwise specified, all reagents were purchased from Sigma-Aldrich (St. Louis, MO, USA). Luria-Bertani (LB) medium and isopropyl-β-D-thiogalactopyranoside (IPTG) were purchased from AMRESCO LLC (Cochran Road Solon, OH, USA). Glycerol-3-phosphate dehydrogenase (α-GDH) and reduced nicotinamide adenine dinucleotide (NADH) were purchased from Roche (Penzberg, Upper Bavaria, Germany). Immobilized Metal Affinity Chromatography (IMAC) resin was purchased from Bio-Rad (Hercules, California, USA). Amicon Ultra 30 kDa filters were purchased from Millipore Corporation (Billerica, Massachusetts, USA).

The sulfhydryl reagents utilized were methylmethane thiosulfonate (MMTS), 5,5′-dithiobis(2-nitrobenzoic acid) (DTNB) and sodium (2-sulfonatoethyl) methanethiosulfonate (MTSES). The last was purchased from Toronto Research Chemicals (Toronto, Canada).

The commercially approved drugs used were omeprazole, rabeprazole and sulbutiamine. The last was purchased from Santa Cruz, Biotechnology (Dallas, Texas, USA).

### Design of the triosephosphate isomerase gene from *E. intestinalis*

The DNA sequence XM_003073826.1 deposited at the NCBI database was used to design the *eitim* gene. Restriction sites for *Nde*I and *BamH*I were added at 5′ and 3′, respectively, to clone the gene into the pET-3aHisTEV expression vector as previously detailed^48^. An *Nde*I internal restriction site at 477 bp was eliminated by changing adenine to guanine at position 479 (A479G). The resulting 752 bp gene was optimized for *Escherichia coli* codon usage. The gene was synthesized by GenScript (Piscataway, NJ) and cloned into the pET-3aHisTEV expression plasmid by using the *Nde*I and *BamH*I restriction sites. The gene encoding EiTIM was completely sequenced to verify the desired sequence and transformed into BL21(DE3)p*Lys*S cells.

### Over-expression and purification of triosephosphate isomerase from *E. intestinalis*

To over-express the protein, the pET-3aHisTEV modified vector was used. This vector contains extra nucleotides at the terminal 5′ region of the inserted gene, encoding 6 histidine (His-tag) residues and a sequence that is recognized by the tobacco etch virus protease (TEVP), which has been demonstrated to facilitate protein purification^48^, at the N-terminal of the protein.

The gene was over-expressed in *E. coli* BL21 DE3p*Lys*S cells. Briefly, 1 L of bacteria culture in LB medium with ampicillin (0.1 mg mL^−1^) was incubated at 37 °C at 180 rpm until it reached an absorbance of 0.8–1 spectrophotometrically measured at 600 nm. Afterward, the culture was induced with 0.4 mM of IPTG and incubated with shaking at 30 °C for 15 h. The induced bacteria were harvested by centrifugation at 5000 rpm for 10 min at 4 °C and suspended in 40 mL of 50 mM Tris pH 8.0 and 50 mM NaCl (lysis buffer) containing 5 mM β-mercaptoethanol and 1 mM phenylmethylsulfonyl fluoride. The bacterial suspension was disrupted by sonication on ice and centrifuged at 9000 rpm for 1 h at 4 °C. To purify the desired protein, IMAC was employed with a Profinity Ni^2+^ charged resin previously equilibrated with lysis buffer. After centrifugation, the clarified fraction was carefully mixed with the equilibrated resin and incubated at 4 °C with shaking for 30 min. To remove the undesired proteins, the resin was subjected to three extensive washes with the same buffer, and the EiTIM was eluted with lysis buffer containing 200 mM imidazole. The eluted fraction was collected and ultrafiltered with centrifugal filtration units (cut-off of 30 kDa) (Merck Millipore, Billerica, Massachusetts). The enzyme was incubated with TEVP to remove the HisTEV-tag, as previously reported^29^. The purity of the recombinant EiTIM was analyzed by sodium dodecyl sulfate-polyacrylamide gel electrophoresis (16% SDS-PAGE) with colloidal Coomassie Brilliant Blue staining. The purified protein was suspended in 100 mM triethanolamine, pH 7.4, 10 mM EDTA (TE buffer) with 50% glycerol and stored at −20 °C until usage. Prior to the assays, the enzyme solution was equilibrated in TE buffer without glycerol and incubated with 5 mM of dithiothreitol (DTT) for 30 min at 4 °C. To remove the reducing agent, the protein was spin filtered in a 1 mL column loaded with Sephadex G-25 Fine Resin from Amersham Biosciences (Amersham, UK) and previously equilibrated with TE buffer^49^. The protein concentration was spectrophotometrically determined at 280 nm, based on the extinction coefficient of EiTIM (ε = 33920 M^−1^ cm^−1^) according to Pace C. *et al*., 1995^50^. Finally, the over-expression and purification of EiTIM was carried out at least five times to ensure its reproducibility.

### Obtaining the kinetic parameters of EiTIM

All the experiments for the functional and structural characterization of the enzyme were performed in triplicate.

The activity of EiTIM was followed in the direction of GAP to DHAP synthesis with a coupled system as previously reported^51^. In brief, stoichiometrically, for each DHAP produced by the enzyme, the coupling enzyme α-glycerol-3-phosphate dehydrogenase (α-GDH) oxidizes one NADH molecule, and the NADH oxidation can be monitored spectrophotometrically at 340 nm. The experimental conditions were 0.9 U of α-GDH, 0.2 mM NADH and GAP concentrations from 0.2 to 10 mM. The GAP was prepared from diethylacetal monobarium according to the manufacturer’s instructions. To measure the initial velocities, 60 ng mL^−1^ EiTIM was gently deposited in the cuvette and kept at 25 °C (due to the easier manipulation and more robust data, most protocols suggest 25 °C as assay temperature)^52^. The activities were plotted as a function of the GAP concentration, and to obtain the kinetic constants, the data were fitted to the Michaelis-Menten equation (1).

1 $$v=\frac{{V}_{max}[{\rm{S}}]}{{K}_{{\rm{m}}}+[{\rm{S}}]}$$

where *v* is the initial velocity, *V*_max_ is the maximum velocity, *K*_m_ is the Michaelis constant (k_−1_ + k_2_)/k_1_, and [S] is the substrate concentration.

### Circular dichroism (CD) profile and melting temperature (Tm) assays

To determine the CD spectrum, the protein was diluted to 0.5 mg mL^−1^ (previously equilibrated in 25 mM phosphate, pH 7.4) and placed in a 0.1 cm quartz cell. The CD spectrum of the sample was recorded from 240 to 190 nm in a Jasco J-810 spectropolarimeter equipped with a thermostated Peltier-controlled cell holder. The spectra were expressed as molar ellipticity (θ), taking into consideration the following equation (2).

2 $$\theta =\frac{{\theta }_{{\rm{o}}{\rm{b}}{\rm{s}}}({\rm{M}}{\rm{R}}{\rm{W}})(100)}{lc}$$

where *θ*_obs_ is the observed mean residue ellipticity in degrees, MRW is the mean residue weight (112.41) of the enzyme in mg mL^−1^, *l* is the light path length in centimeters, and *c* is the concentration.

The thermal denaturation of this protein sample was measured by recording its CD spectrum at 222 nm. The sample was subjected to temperature increases from 20 to 90 °C at a rate of 1 °C min^−1^. From the experimental data, the apparent fraction of denatured protein was calculated, and the melting temperature (Tm) was determined, as previously reported^32^.

### 8-Anilinonaphthalene-1-sulfonic acid (ANS) fluorescence assays

The fluorescence of the probe 8-anilinonaphthalene-1-sulfonic acid (ANS) was monitored in the presence of EiTIM. The experimental conditions were as follows: 0.5 mg mL^−1^ of the protein was incubated in the absence or presence of 0.1 mM ANS (solubilized in methanol). The fluorescence spectra were recorded in an LS 55 fluorescence spectrometer (Perkin Elmer, Waltham, MA, USA). The samples were excited at 395 nm, and the fluorescence emission spectrum was recorded from 400 to 600 nm at 25 °C with a scanning rate of 200 nm min^−1^. The background fluorescence signal of the TE buffer with ANS was subtracted from the sample reading. The results were expressed in arbitrary units (a.u.) of fluorescence intensity *versus* wavelength.

### Functional and structural assays of EiTIM in the presence of sulfhydryl reagents

To determine the inactivation behavior of the enzyme, it was incubated in the presence of sulfhydryl reagents. EiTIM (0.5 mg mL^−1^) was incubated separately with MMTS, MTSES and DTNB (dissolved in TE buffer) at concentrations ranging from 0 to 200 μM over 2 h at 37 °C. At the end of the incubation period, an aliquot was withdrawn and diluted, and the residual activity (with 1 mM GAP) was measured by following the oxidation of NADH at 340 nm.

Additionally, the structural parameters of the enzyme derivatized with the sulfhydryl reagents were evaluated. Briefly, the CD spectrum and Tm value were obtained for EiTIM (2 mg mL^−1^) previously incubated with 0.5 mM of MMTS, MTSES or DTNB for 2 h at 37 °C. To remove excess reagent, the inactivated enzyme was ultrafiltered with Centricon tubes (30 kDa cut-off), and the protein concentration was recalculated to determine the molar ellipticity and Tm, as previously mentioned. Additionally, the extrinsic fluorescence levels of the untreated enzymes (control) and the enzymes treated with the sulfhydryl reagents were determined. Briefly, the fluorescence emission spectra (from 400 to 600 nm) of the control and derivatized enzymes were monitored with excitation at 395 nm. Then, 0.1 mM of ANS was carefully added to each sample, and the fluorescence emission spectrum was recorded. Finally, ANS in TE buffer was measured, and the background signal was subtracted. The results were expressed in term of arbitrary units (a.u.) of fluorescence intensity *versus* wavelength (nm).

### Quantification of free Cys in EiTIM treated with sulfhydryl reagents

To determine the number of Cys residues derivatized in EiTIM, assays were performed with 2 mg of protein previously incubated without (control) or with each of the sulfhydryl reagents at 0.5 mM for 2 h at 37 °C. After incubation, an aliquot was taken to measure the residual activity of the recombinant protein.

The content of free Cys was quantified as follows. Excess sulfhydryl reagents were removed by ultrafiltration with filtration units, and the concentration of the protein was recalculated at 280 nm, as described above. The free Cys content of the samples was spectrophotometrically determined in the presence of 1 mM DTNB and 5% sodium dodecyl sulfate (SDS) dissolved in TE buffer by following the increase in absorbance at 412 nm (ε _412 nm_ = 13.6 mM^−1^ cm^−1^)^53^. The number of derivatized Cys residues was calculated by subtracting the free Cys of the derivatized enzyme from the free Cys of the control enzyme.

### Assays of recombinant EiTIM with approved drugs

We performed assays to explore the possibility of inactivating the EiTIM by using existing approved commercial drugs. The drugs assayed were omeprazole, rabeprazole and sulbutiamine.

To perform acid activation of the omeprazole, a stock solution of 200 mM omeprazole in DMSO was incubated with 10% (v/v) of 0.1 M HCl in dark conditions for 3 h at room temperature^34^. This solution was then diluted one thousand times in TE buffer, and the resulting dilution (0.2 mM) was used in the experiments. Rabeprazole and sulbutiamine were directly solubilized in TE buffer at 20 mM and diluted to 0.2 mM. The assays were performed by incubating 0.5 mg mL^−1^ of the enzyme for 2 h at 37 °C with increasing concentrations of the drugs (0 to 50 μM). After the incubation period, the enzymatic activity was measured by withdrawing an aliquot of each experimental condition, as described above. The results were expressed in terms of the percentage of activity *versus* the drug concentration.

## Electronic supplementary material

Supplementary Information File #1
