# Supplementary material for: First characterization of a microsporidial triosephosphate isomerase and the biochemical mechanisms of its inactivation to propose a new druggable target
Source: Sci Rep. 2018 Jun 5;8:8591. doi: 10.1038/s41598-018-26845-z (PMC5988755; doi:10.1038/s41598-018-26845-z)
Supplement: Supplementary file 1 — Supplementary Information File #1 [file 41598_2018_26845_MOESM1_ESM.pdf]

First characterization of a microsporidial triosephosphate isomerase and the biochemical mechanisms of its inactivation to propose a new druggable target.

Itzhel García-Torres, Ignacio De la Mora-De la Mora, Gloria Hernández-Alcántara, Dora Molina-Ortiz, Silvia Caballero-Salazar, Alfonso Olivos-García, Gabriela Nava-Balderas, Gabriel López-Velázquez, Sergio Enríquez-Flores.

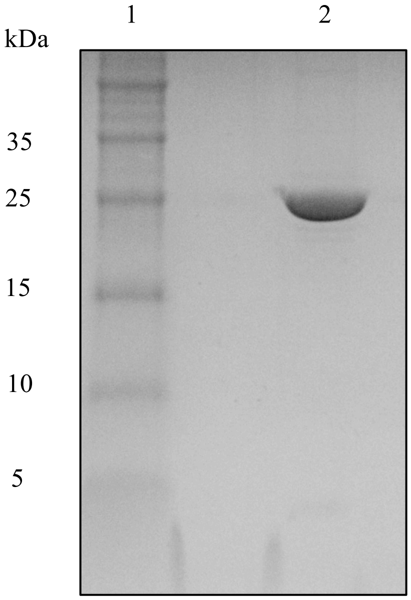

Supplementary Fig. S1. Recombinant EiTIM purified. SDS-PAGE (16 %) shows the purification of the protein. Molecular masses are shown to the left in kDa; Lane 1, molecular marker; Lane 2, EiTIM purified and without HisTEV-tag.

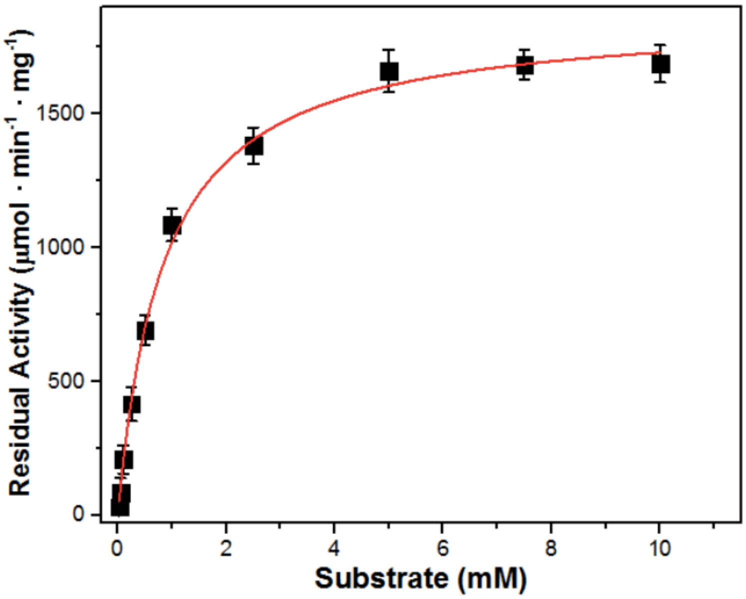

Supplementary Fig. S2. Michaelis-Menten plot obtained for the EiTIM enzyme saturated with substrate (GAP). For the enzymatic assays, the protein was incubated at 0.5 mg mL^{-1} at 25 ºC, and an aliquot was taken and diluted to measure the residual activity. The substrate concentration was varied from 0 to 10 mM.

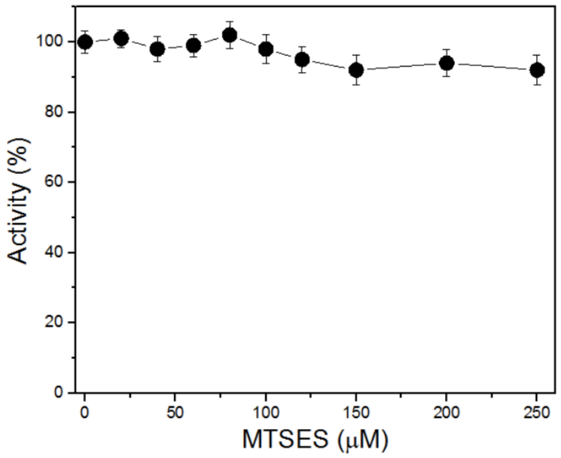

Supplementary Fig. S3. Determination of HsTIM activity after exposure to the sulfhydryl reagent MTSES. The protein was incubated at increasing concentrations of MTSES (closed circles). After 2 h of incubation at 37 ºC, an aliquot was withdrawn, and the residual activity was determined as reported in Materials and Methods.

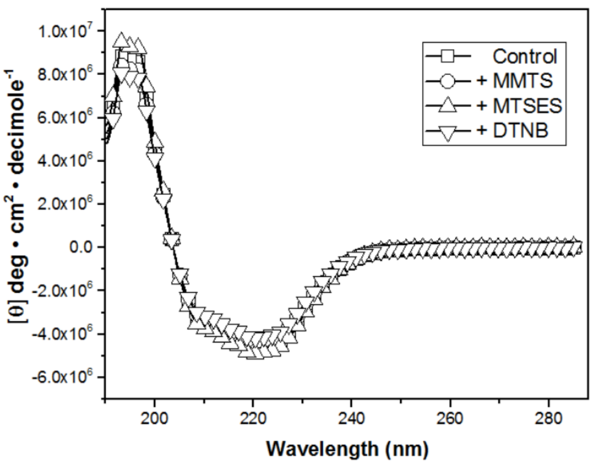

Supplementary Fig. S4. **Far-UV CD spectra of non-derivatized EiTIM and EiTIM derivatized with sulfhydryl reagents**. The CD spectrum (280 to 190 nm) was measured with 0.5 mg mL^{-1} of the protein at 25 ºC.

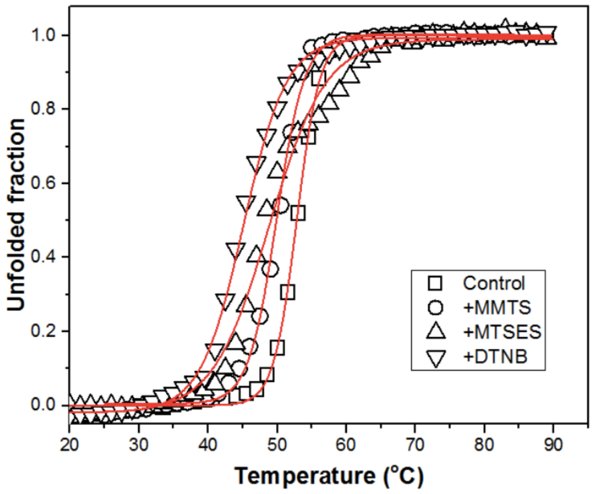

Supplementary Fig. S5. **Thermal denaturation of non-derivatized EiTIM and EiTIM derivatized with sulfhydryl reagents.** The CD signal of the protein (0.5 mg mL^{-1}) was measured with increasing temperature at 222 nm. The red line corresponds to the Boltzmann adjustment, and the box within the graph indicates the EiTIM conditions.
